# Application of the Threshold of Toxicological Concern (TTC) in Food Safety: Challenges and Opportunities

**DOI:** 10.3389/ftox.2021.655951

**Published:** 2021-03-19

**Authors:** Rositsa Serafimova, Tamara Coja, George E. N. Kass

**Affiliations:** ^1^European Food Safety Authority, Parma, Italy; ^2^Austrian Agency for Health and Food Safety, Vienna, Austria

**Keywords:** risk assesment, flavorings, EFSA, pesticides, food contact material

## Abstract

The safety assessment of chemicals added or found in food has traditionally made use of data from *in vivo* studies performed on experimental animals. The nature and amount of data required to carry out a risk assessment is generally stipulated either in the different food legislations or in sectoral guidance documents. However, there are still cases where no or only limited experimental data are available or not specified by law, for example for contaminants or for some minor metabolites from active substances in plant protection products. For such cases, the Threshold of Toxicological Concern (TTC) can be applied. This review explores the use of the TTC approach in food safety in the European Union, in relation to the different food sectors, legal requirements and future opportunities.

## Introduction

Regulatory risk assessment of chemicals added or found in food and feed, has traditionally made use of data from *in vivo* studies performed on experimental animals. Such studies aim at identifying and characterizing the potential of chemicals for inducing adverse effects following short or prolonged exposure, directly or through exposure *in utero*. *In vivo* studies also provide critical information on how chemicals are handled by the body, in terms of their absorption, distribution, metabolism and excretion (ADME).

This type of studies is generally requested by risk assessment bodies for the safety evaluation of chemicals to be put on the European market *de novo* or that are in need of a re-evaluation. This is because of legislation or of the need to conform to internationally agreed standards. However, for chemicals not intentionally added, but that could be found in food, such as contaminants, chemicals produced from (bio)degradation-(bio)transformation processes or as unintended reaction by-products or impurities in the manufacturing process, often little or no experimental toxicological information is available (mainly because there is no such detailed data requirements in the legislation). Many of them occur in low or very low concentrations, and often, we have only become aware of their presence over the past decade thanks to the progress in analytical techniques. For addressing the potential effects of these data-poor chemicals on human health, the use of alternative to animal tests approaches is often recommended.

## Food Safety in the European Union

In the European Union (EU), the food sector is strictly controlled by legislation when it comes to the safety of chemicals intentionally added to food, plant protection products, food contact materials, novel foods, GM products, and for a substantial number of contaminants. The General Food Law Regulation (Regulation (EC) No 178/2002) lays down the general principles, requirements and procedures that underpin decision making in matters of food and feed safety, covering all stages of food and feed production and distribution. The same Regulation also sets up the European Food Safety Authority (EFSA) as an independent agency responsible for scientific advice and support.

While the different sectoral EU legislations pertinent to food safety all aim at protecting the consumer in general, they vary considerably as to the level of details provided in the legislative text as to the type and depth of information to be provided to EFSA for performing a chemical risk assessment. For instance, the legislation addressing plant protection products (Commission Regulation (EU) No 283/2013, Commission Communication 2013/C 95/01) is rather prescriptive for the types of studies that need to be performed to support a dossier whereas other sectors, such as food additives, the legislation (Regulation (EC) No 1333/2008) simply states that the food additives must be safe when used. For such cases, EFSA develops appropriate guidance documents to inform what types of data are needed for a risk assessment (EFSA, 2012a).

Overall, the type and quantity of safety information requested from industry is dependent on the sector, and broadly correlates with the anticipated or measured use or exposure levels. Thus, for a risk assessment of a food additive, where exposure can be high, a complete set of data ranging from ADME, genotoxicity to repeated dose toxicity studies, including reproductive and developmental toxicity studies are requested by EFSA, unless there are specific conditions, such as negligible bioavailability, where a more limited set of data is required (EFSA, 2012a). In contrast, for a food contact material with very low migration potential (e.g., <50 μg/kg food), and hence low exposure of the consumer, only the lack of genotoxic potential needs to be demonstrated (EFSA, 2008). Furthermore, all studies required for a regulatory risk assessment must be performed according to the OECD test guidelines and should follow the OECD principles of Good Laboratory Practice (GLP) conditions (in accordance with the rules set out in the Directive 2004/10/EC).

Where there is no identifiable producer or commercial association behind a chemical, as in the case of many contaminants, especially those that occur naturally, such as mycotoxins and some heavy metals, the type, quality and quantity of data available for a risk assessment may be highly variable. Likewise, for some minor metabolites from plant protection products to which the consumer may be exposed to following their formation through the biotransformation of the active substance in plants, livestock or soil, there may be insufficient data to conduct a full risk assessment. This is especially the case where they cannot be synthetized for testing purposes.

## The TTC Approach in Chemical Risk Assessment

The Threshold of Toxicological Concern (TTC) is an approach developed to address the situation where the structure of a compound is known, exposure to that compound is low but where there is insufficient compound-specific toxicity data to enable a risk assessment (Munro et al., 1996; Barlow, 2005). The TTC approach was first proposed by Munro and co-workers in 1996, and it defines a threshold of exposure level below which the risk to human health is assumed negligible. The TTC approach uses the Cramer classification of chemicals (Cramer et al., 1978), which places them into one of three structural classes based on their structural complexity. Munro and co-workers used a database containing subchronic and chronic animal studies of 613 chemicals representing a range of industrial chemicals, pharmaceuticals, food chemicals, environmental, and consumer chemicals. For each class, the 5th percentile of the lognormal cumulative distribution of the No-Observed-Effect-Levels (NOELs) (to which a 100-fold safety factor was added) was computed to derive the human exposure threshold values, known as TTC values. The human exposure thresholds for these structural classes are 1,800, 540, and 90 μg/person/day, respectively (Table 1).

**Table 1:** TTC values – classification of chemicals.

**Classification**	**TTC value in μg/person per day**	**TTC value in μg/kg bw per day^a^**
Potential DNA-reactive mutagens and/or carcinogens	0.15	0.0025
Organophosphates and carbamates	18	0.3
Cramer Class III	90	1.5
Cramer Class II	540	9.0
Cramer Class I	1,800	30

a *The conversion to μg/kg bw per day used a body weight value of 60 kg as done originally by Munro and co-workers to derive the generic human exposure threshold values (Munro et al., 1996). It should be noted that EFSA's current recommendation is to use a default value of 70 kg, when appropriate, for adult body weight (EFSA, 2012b)*.

For a comprehensive review of the development of the TTC and its history and applicability to chemical risk assessment, the reader is referred to the many excellent reviews on the subject published elsewhere (Bhatia et al., 2015; Boobis et al., 2017; Feigenbaum and Worth, 2019) or as part of this special issue. The TTC approach has also been adapted to non-oral routes of exposure (e.g., Safford, 2008; Tluczkiewicz et al., 2016; Williams et al., 2016; Hoersch et al., 2018) but these are not discussed further in this review.

## Application of the TTC Approach in Food Safety Assessment in the EU: An Overview

The use of the TTC approach in the food safety assessment in EU needs to be contextualized with the data requirements for chemicals in the different sectors (Table 2). The TTC approach should not be used for chemicals for which the European Union (EU) food legislation requires the submission of toxicity data (EFSA, 2019c). Furthermore, for food safety assessments, when data are available for a risk assessment, these data should be used and not the TTC approach.

**Table 2:** Sectors and areas of use of the TTC approach or TTC threshold-based safety values.

**Sector**	**Specific use of TTC**	**References**
Flavoring substances in food	Safety assessment of flavorings	EFSA, 2010
Food additives	Impurities, metabolites and degradation products of food additives	EFSA, 2012a
Contaminants	Pharmacologically active substances in food of animal origin	EFSA, 2018
Plant protection products	Some metabolites and degradation products of plant protection products in the context of residue definition for risk assessment	EFSA, 2016a
Flavoring additives in feed	“Maximum acceptable feed concentrations” for flavoring additives from default values for feed consumption	EFSA, 2017
Safety of recycling processes for recycled plastics used in Food Contact Materials	Criteria for the safety evaluation of mechanical processes to produce recycled poly(ethylene terephthalate) (PET) for materials and articles in contact with food	EFSA, 2011

Consequently, the TTC approach is used in the EU as a tool in food safety assessment only in a limited number of areas (see below). This is the case of flavoring substances, where the TTC approach is applied to the substance when evidence of its lack of genotoxicity potential has been demonstrated. Likewise, for substances related with the “principal” substance, such as unavoidable impurities or (bio)degradation products, the TTC approach has supported the risk assessment process in the case of active substances in plant protection products and food additives. Moreover, in several other areas of food safety, such as food contact materials or unavoidable contaminants, one or more thresholds of the TTC approach have served as a basis for safety thresholds in the safety assessment or as triggers for the need of experimental data.

### Flavoring Substances

The area of food flavorings was the first and remains one of the main areas where the TTC approach is used in food safety assessment. In its 44th report, the Joint FAO/WHO Expert Committee on Food Additives (JECFA) reported in 1995 that for “*those flavoring agents currently in use for which no toxicity or metabolic data exist, but where intake is extremely low, it might be possible to specify a threshold below which intake is considered safe (human exposure threshold)*.” (FAO and WHO, 1995). The approach referred to in this document is that of the TTC developed by Munro and co-workers, involves the subdivision of flavorings into the three Cramer structural classes (I, II, III) but precedes the publication of their seminal paper. The decision to use the TTC approach for the safety evaluation of flavoring substances was a pragmatic one: the need of safety assessment of the roughly 1,200 flavoring substances used at the time with no or limited toxicological and metabolic data, but extremely low human exposure. The decision tree approach for the assessment of flavorings presented at that meeting was subsequently modified by the Committee and presented at the 46th report in 1997 (FAO and WHO, 1997).

The Scientific Committee on Food (SCF) of the European Commission assessed the JECFA approach to use the TTC approach for the safety evaluation of flavoring substances. They considered that “this procedure is a pragmatic approach” and, in principle, that the SCF is “prepared to use this approach for chemically defined flavoring substances within the evaluation programme of the Commission.” (Scientific Committe on Food, 1999) (see also Commission Regulation (EC) No 1565/2000). However, it should be noted that the SCF and JECFA both recommended that the intake estimation for flavoring agents needed further development. Furthermore, flavoring substances should also be examined for structural alerts of potential genotoxicity.

When the responsibilities of the SCF were transferred to the European Food Safety Authority in 2002, the TTC approach developed by the JECFA and endorsed by the SCF remained the principal tool for the safety assessment of flavoring substances [see e.g., (EFSA, 2004)]. No toxicity data were required for flavoring substances devoid of structural alerts for genotoxicity (in the parent molecule or in predicted metabolites) and when the estimated daily intake of the substance was lower than its corresponding TTC value. By 2010, EFSA had evaluated over 2,000 of the around 2,500 flavoring substances used in the European Union by implementing this approach; another 400 substances remained in need of additional experimental data for the finalization of their evaluation. In 2010, EFSA developed a guidance document for the risk assessment of flavoring substances in and on foods (EFSA, 2010) in which, based on the experience gained during the previous evaluations, the TTC approach is embedded as part of the procedure to be followed. Around the same time, EFSA initiated a more general assessment of the potential use of the TTC in food safety (EFSA, 2012c) and, more recently, a guidance on the use of the TTC approach in food safety, including a new decision tree, was published (EFSA, 2019c). A similarly updated TTC decision tree approach for flavoring substances was published by the JECFA in 2016 (FAO and WHO, 2016).

The use of the TTC approach is not limited to flavorings intended for human consumption but also when added to feed to define “maximum acceptable feed concentrations” for flavoring additives based on default values for feed consumption (EFSA, 2017).

### Food Contact Materials

The safety evaluation of substances in food contact materials in the EU uses a thresholded approach that has in its origin the Threshold of Regulation (TOR) concept of 1.5 μg/person/day. The TOR concept was introduced in the US FDA policy in 1995 as a process for dealing with components of food contact materials that pose a negligible risk (Food and Drug Administration, 1995). The TOR threshold was set at a dietary concentration of 0.5 parts per billion (ppb) after considering the dietary levels at which toxic effects are observed in both chronic and short-term animal feeding studies (Cheeseman, 2005). The database that served to establish the TOR was the Carcinogenic Potency Database (CPDB) compiled by Gold between 1984 and 1997 and comprising 477 substances (Gold et al., 2005) and carcinogenicity as the most sensitive toxicological endpoint. Therefore, the basis to the TOR is distinct to that of the TTC, and hence the two approaches are similar but not identical.

The SCF adapted the TOR approach (Scientific Committe on Food, 2001) by considering thresholds of migration of the chemical into food, and hence the level of potential exposure. These thresholds of migration are used to define how extensive the toxicological dataset needs to be submitted rather than providing safety levels. More recently, three threshold levels of human exposure, namely 1.5, 30, and 80 μg/kg bw per day, as triggers for the requirement of toxicity data in addition to genotoxicity data were proposed (EFSA, 2016b). The first two thresholds of 1.5 and 30 μg/kg bw per day originate from the TTC concept. For chemicals with a calculated exposure up to 1.5 μg/kg bw per day, and for chemicals which belong to Cramer Class I and to which exposure is <30 μg/kg bw per day, no additional toxicity studies are required, as long as they are not subject to the TTC exclusion criteria (Table 3). The third exposure threshold of 80 μg/kg bw per day sees its origin in previous SCF guidelines (Barlow, 1994); exposure above this level is considered to approach that observed for food additives and would lead to a request of a corresponding toxicological data set (EFSA, 2016b).

**Table 3:** Exclusion categories where the TTC approach is not applicable.

**Chemicals which are not represented in the database or are outside the domain of applicability**
• Inorganic chemicals • Proteins • Nanomaterials • Radioactive chemicals • Organosilicon chemicals • Metals in elemental, ionic or organic form. However, in the case of organic salts, where the counter ion is an essential metal (e.g., sodium), the recommendation is that the TTC approach could be applied to the organic ion.
**Chemicals with special properties**
• High potency carcinogens: aflatoxin-like, azoxy- or *N*-nitroso chemicals and benzidines • Steroids • Chemicals with a potential for bioaccumulation. This includes chemicals like polyhalogenated-dibenzodioxins, -dibenzofurans, and -biphenyls.

Chemicals used in the manufacture of plastics may contain contaminants originating from their manufacture and may form reaction and degradation products that are not oligomers as a result of the production or treatment process of the food contact material. The safety evaluation of these so-called non-intentionally added substances (NIAS) is complex, and where the structure is known, the use of the TTC has been suggested as a possible approach (EFSA, 2016b).

The TTC concept was also used for the development of the criteria for the safety evaluation of mechanical recycling processes for poly(ethylene terephthalate) (PET) intended for use in materials and articles in contact with food (EFSA, 2011). The approach used by EFSA was to take into account a worst-case scenario contaminants potentially present in post-consumer PET used as input into a recycling process and that any dietary exposure to these contaminants in recycled PET should be below 0.0025 μg/kg bw/day (the threshold for DNA-reactive mutagens and/or carcinogens, Table 1) (Kroes et al., 2004; EFSA, 2012c). This would ensure that any unknown contaminant possibly present is treated in a conservative way (i.e., as a genotoxic chemical) and is sufficiently removed by the recycling process under evaluation.

### Food Additives

The risk assessment of food additives always involves the use of safety data from *in vivo* and *in vitro* toxicity studies. An exception may be some impurities, metabolites and degradation products of the food additive. For unavoidable genotoxic residuals, for which carcinogenicity data are not available, the TTC value of 0.15 μg/person/day (0.0025 μg/kg bw/day) would be considered because of the high probability of protection against carcinogenic genotoxic effects, which would also cover heritable effects (EFSA, 2012a).

### Non-allowed Pharmacologically Active Substances Present in Food of Animal Origin

As a result of the treatment of food-producing animals with veterinary medicinal products, residues of pharmacologically active substances can be present in animal products intended for human consumption. In accordance with Regulation (EC) No 470/2009, these veterinary medicinal products may only be placed on the market if the residues in animal products do not pose any harm to the consumer. Pharmacologically active substances fulfilling this condition are classified as “allowed substances” (Regulation (EU) No 37/2010). All other pharmacologically active substances are considered as “non-allowed substances,” with a specific subgroup termed “prohibited substances.” EFSA recently issued a guidance document for establishing Reference Points for Action (RPAs) for non-allowed pharmacologically active substances present in food of animal origin (EFSA, 2018).

For the specific case of non-allowed pharmacologically active substances for which there is either direct evidence of genotoxicity or insufficient evidence to conclude that it is not genotoxic, the TTC value of 0.0025 μg/kg bw per day was identified as a toxicological screening value (TSV). It should be noted that the database used by Munro and co-workers to develop the TTC approach is limited to a small number of pharmacologically active substances and, consequently, the other TTC values were not applied for non-allowed pharmacologically active substances present in food of animal origin.

### Metabolites and Degradation Products of Active Substances in Plant Protection Products

While for active substances in plant protection products very extensive toxicological data requirements are defined (Commission Regulation (EU) No. 283/2013), this is not the case for pesticides metabolites, formed in plants, livestock or in the environment. The TTC concept is proposed to be used in the assessment of metabolites found in food commodities of plant and animal origin for which chemical-specific toxicological data are not available (EFSA, 2016a). The TTC approach is used as a decision point for requesting experimental data when the exposure to the metabolites exceeds the relevant TTC values at the time point relevant for consumption. It should be noted that the EFSA guidance proposes for the exposure that all identified but toxicologically non-characterized metabolites are summed up and compared to their specific TTC value. An exception would be in case of a scientifically justified assumption of different toxicological profiles among the metabolites. The Guidance refers to thresholds of 0.3 μg/kg bw per day (for organophosphates and carbamates pesticides) or 1.5 μg/kg bw per day (Cramer Class III and Cramer Class II) and 30 μg/kg bw per day (Cramer Class I) for chronic assessment (EFSA, 2012c, 2019c).

When the TTC approach is applied to pesticides metabolites, the potential exposure from all possible sources (e.g., environmental background, biocidal uses, veterinary medicinal products) should be considered to ensure that the total exposure of consumers to the pesticide metabolites is assessed appropriately. In the case of groundwater, non-relevant metabolites with no toxicological data are assessed following the US FDA TOR of 1.5 μg/person/day (European Commission, 2003). Consequently, a situation may arise where the same metabolite will be assessed against different thresholds if found in both groundwater and some food commodities. Here, further harmonization is urgently needed.

## EFSA Guidance on the Use of the TTC Approach in Food Safety Assessment

Recently, a generic EFSA Guidance was developed to provide practical help in the appropriate use of the TTC approach across the different sectors of chemical risk assessment within EFSA's remit (EFSA, 2019c). The aim was to build on the latest scientific developments in the TTC approach and to provide a decision tree that is broadly applicable for a general use of the TTC approach in food chemical risk assessment.

**Figure 1:**
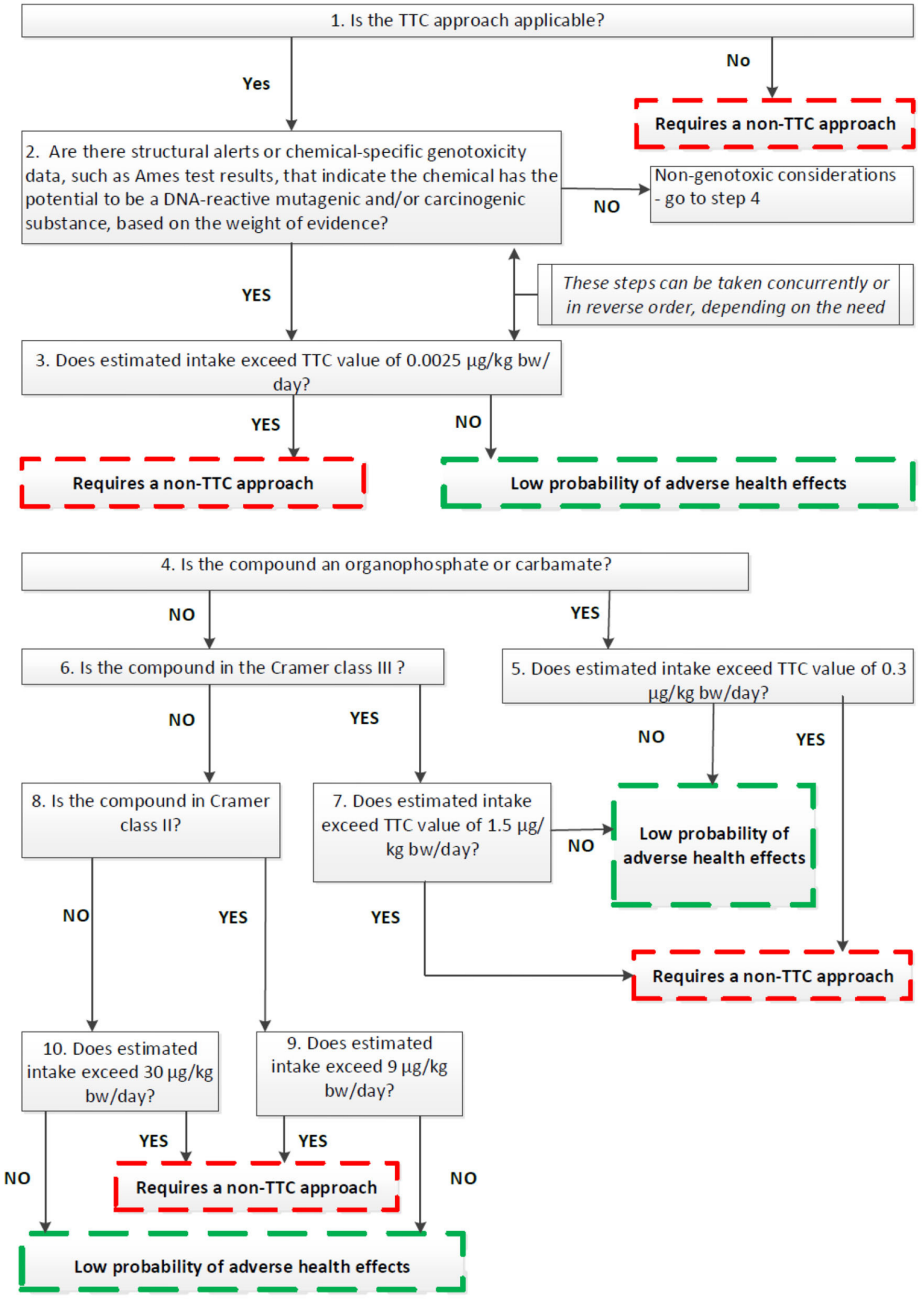
EFSA's decision tree for the use of the TTC approach in food safety (EFSA, 2019c).

The EFSA Guidance builds on the general principle that the TTC approach can be applied to any chemicals with known structure and for which oral exposures can be evaluated and where toxicity data are scarse. The approach also makes use of defined chemical exclusion categories (Table 3) for which TTC should not be applied. In the EU, the TTC approach should not be applied to chemicals for which EU legislation for food or feed requires the submission of toxicity data. The application of the TTC approach is illustrated in Figure 1 in the form of a decision tree that illustrates a stepwise way of proceeding.

In general, the TTC approach can be used to cover the whole population. However, in the case of infants below the age of 4 months and young children, special considerations need taking into account because infants, in contrast to adults, are not only considered to be more sensitive to some toxicological insults but also have a higher food intake per kilogram body weight (EFSA, 2019c). Another area calling for special considerations is in the case of mixtures of chemicals where the applicability of the TTC approach depends on the nature and the level of characterization of the mixture. For mixtures with a full chemical characterization, the TTC approach can be applied using the assumption of dose addition (EFSA, 2019b). For mixtures with a not fully defined chemical composition, the use of the TTC approach may be justifiable on a case-by-case basis providing that there is enough information (or analysis) to demonstrate that the mixture does not contain chemicals listed in the exclusion categories. Further considerations to be made are whether there is sufficient evidence to exclude the presence in the mixture of DNA-reactive components, or organophosphates or carbamates. In such case, the mixture could be considered in Cramer Class III. However, in the absence of such evidence, the unknown components may be managed as potentially DNA-reactive and therefore the sum of these (mixture) components should be below the TTC value of 0.0025 μg/kg bw (EFSA, 2019a,b,c).

## Testing the TTC Values Using the EFSA's OpenFoodTox Database

The level of protection provided by the TTC values for chemicals under EFSA remit was analyzed recently using EFSA's OpenFoodTox database (Reilly et al., 2019). EFSA's OpenFoodTox chemical hazards database contains all the chemicals for which EFSA is responsible for their chemical risk assessment, includes pesticides, food additives, flavorings and nutrient sources, feed additives and contaminants. After elimination of the chemicals with a structural alert or empirical evidence for genotoxicity, or belonging to the organophosphate or carbamate groups or part of the exclusion categories for the TTC approach, the analysis of the remaining 329 chemicals provided threshold values of 1,000 μg/person per day (90% confidence interval: 187–2,190) and 87 μg/person per day (90% confidence interval: 60–153) for Cramer Classes I and III, respectively, compared to the corresponding original threshold values of 1,800 and 90 μg/person per day (Reilly et al., 2019). This confirms the protectiveness of the TTC values for Cramer Classes I and III to chemicals relevant for food safety. Due to only few chemicals falling into Cramer Class II, this class was excluded from our analysis.

## Challenges and Opportunities in the Use of the TTC in Food Safety

An ongoing discussion has been the adequacy of the Cramer classification scheme (Dewhurst and Renwick, 2013; EFSA and WHO, 2016). Although proposals for improvements have been made [see e.g., (Tluczkiewicz et al., 2011)], the Cramer classification scheme as used in the TTC approach remains conservative and therefore protective of human health (EFSA, 2019c). To avoid the shortcomings of the Cramer classification, the US-FDA has developed the Expanded Decision Tree (EDT) that screens and prioritizes chemicals for safety testing according to their toxic potential using a sequence of structure-based questions and assigns the chemical to one of six EDT Classes [cited in (EFSA, 2019c)]. Each class has a Threshold of Toxicological Concern (TTC) level associated with it.

The TTC value of 0.0025 μg/kg bw is aimed at potential genotoxic chemicals that are DNA-reactive mutagens. However, this does not cover all mechanisms of genotoxicity such as clastogenicity (structural chromosomal aberrations) or aneugenicity (numerical chromosomal aberrations). To address chemicals that are aneugenic and can induce aneuploidy, EFSA has recently, developed and put for public consultation a draft guidance on aneugenicity assessment in which the applicability of the TTC concept is also discussed (EFSA, 2020). In theory, for chemicals without concern for gene mutations and clastogenicity and with no or insufficient toxicological data, the TTC approach could be applied on condition that a point of departure for aneugenicity can be identified. The entry point in the TTC decision tree for such chemicals would be at step 4 (Figure 1). The relevant TTC value to be applied to allow for the absence of toxicological date should be at least 100-fold lower than the point of departure for aneugenicity. In case a point of departure for aneugens cannot be established, TTC approach is not recommended. However, this approach needs further assessing for its applicability in food safety since currently there is insufficient information on chemical structures leading to aneugenicity and limited representation of pure aneugens in the TTC databases. It should be noted that at the moment of preparation of this publication the above mentioned Guidance has not been adopted by the EFSA Scientific Committee and the final text might be subject of further amendments (to be finalized Summer 2021).

Another limitation for the TTC approach is that it uses toxicological data from oral doses administered to experimental animals to estimate an equivalent human external exposure. A refinement of the TTC approach would be to take in consideration the internal exposure and link it to the internal concentration at the target leading to the occurrence of adversity. The use of an internal TTC has been proposed by several researchers (Partosch et al., 2015; Ellison et al., 2019; Blackburn et al., 2020), and if successful, this development could provide a more precise way of assessing various sources of uncertainty associated with the TTC. However, the prediction of an internal exposure for chemicals with no or very limited data may prove challenging.

Finally, when the TTC approach is applied one of the biggest practical challenge remains the accurate calculation of the relevant exposure. The two factors which need to be addressed are the consideration of all possible sources of exposure for one chemical (e.g., environmental background, biocidal uses, veterinary medicinal products) and the combined exposure to multiple chemicals with similar toxicological impact on human health. These two issues in many cases hinder the application and acceptance of the TTC approach for regulatory purposes, and, therefore, would benefit from further work.

## Author Contributions

GK, TC, and RS contributed equally to the design, writing, and final editing of the paper. All authors contributed to the article and approved the submitted version.

## Conflict of Interest

The authors declare that the research was conducted in the absence of any commercial or financial relationships that could be construed as a potential conflict of interest.
